# Transcriptome and protein interaction profiling in cancer cells with mutations in histone H3.3

**DOI:** 10.1038/sdata.2018.283

**Published:** 2018-12-11

**Authors:** Jinyeong Lim, Joo Hyun Park, Annika Baude, Jörg Fellenberg, Jozef Zustin, Florian Haller, Irene Krücken, Hyun Guy Kang, Yoon Jung Park, Christoph Plass, Anders M. Lindroth

**Affiliations:** 1 Graduate School of Cancer Science and Policy, Cancer Biomedical Science, National Cancer Center, Gyeonggi-do, Republic of Korea; 2 Metabolism and Epigenetics Laboratory, Department of Nutritional Science and Food Management, Ewha Womans University, Seoul, Republic of Korea; 3 Division of Epigenomics and Cancer Risk Factors, German Cancer Research Center, Heidelberg, Germany; 4 Research Center for Experimental Orthopedics, Clinic for Orthopedic and Trauma Surgery, University of Heidelberg, Heidelberg, Germany; 5 Institute of Osteology and Biomechanics, University Medical Center Hamburg Eppendorf, Hamburg, Germany; 6 Institute of Pathology, University Hospital Erlangen, Erlangen, Germany; 7 Institute of Pathology, University of Leipzig, Leipzig, Germany

**Keywords:** Cancer epigenetics, Genetics research

## Abstract

Mutations of histone variant H3.3 are highly recurrent in childhood glioblastoma and in young adults with Giant Cell Tumor of the Bone (GCTB). The heterozygotic representation of the mutations in the tumors, and with potential histone H3 and H3.3 redundancy, suggest that the mutations are gain-of-function by nature. To address common H3.3 point mutations, we have generated data from GCTB patient samples with H3.3 G34W substitutions and engineered human GFP-tagged H3.3-mutated isogenic cell lines for high throughput data comparisons. First, a total of thirty-six patient samples and cell lines were used to acquire gene expression transcriptome data using microarray and RNA-sequencing. The expression data were validated with the orthogonal nCounter assay. Second, to uncover the H3.3-GFP interaction proteomes from the isogenic cell lines, immunoprecipitation of unmutated wild type, K27M, G34R, and G34W substitutions were performed. The RNA-sequencing data and the H3.3 interaction proteome enable potentially important functional insight into the tumorigenic process and should spur further detailed analysis.

## Background & Summary

Recurrent genomic alterations are a cornerstone in cancer development and reproducible mutational profiles have been observed at defined genomic locations. Whole genome sequencing of tumors from adults and seniors have uncovered genomic landscapes with hundreds of small and large-scale mutations, revealing the complexity of chromosomal rearrangements influencing tumorigenesis^1^. In contrast, tumors from children and young adults develop from a less complex cytogenetic background, simplifying the process of uncovering the driving forces of tumorigenesis and their transcriptional profiles.

This is exemplified by the recent discovery of recurrent mutations of histone H3 and the replication-independent variant H3.3 in pediatric Glioblastoma^2^ and Giant Cell Tumor of the Bone (GCTB)^3^ where very few other genic mutations were discovered after whole-exome sequencings. Yet, the histone mutations affected global epigenetic modifications of important residues, dramatically changing gene expression of targeted genes, seemingly driven by the histone mutations that lead to amino acid substitutions at or very near frequent post-translational modified amino acids; namely the K27M, G34R/V/L/W, and K36M substitutions^3^.

The power behind these histone mutations can be traced to their role as pre-eminent binding sites for proteins influencing transcription. The first, and perhaps the best example, is the H3.3 K27M substitution leading to binding and catalytic inhibition of the polycomb repressive complex 2 (PRC2), with global loss of H3K27 methylation across the genome as a result^4,5^. A similar example is the H3.3 K36M substitution binding the enzymes MMSET and SETD2, leading to global reduction of H3K36 methylation^6^. These two examples indicate that mutations of the N-terminal tail of histones can acquire novel binding properties and greatly influence transcription. We address the possibility that the cancer-related histone H3.3 substitutions in this study bind yet unknown proteins, allowing them to be purified and characterized by gel separation and mass spectrometry.

**Figure 1:**
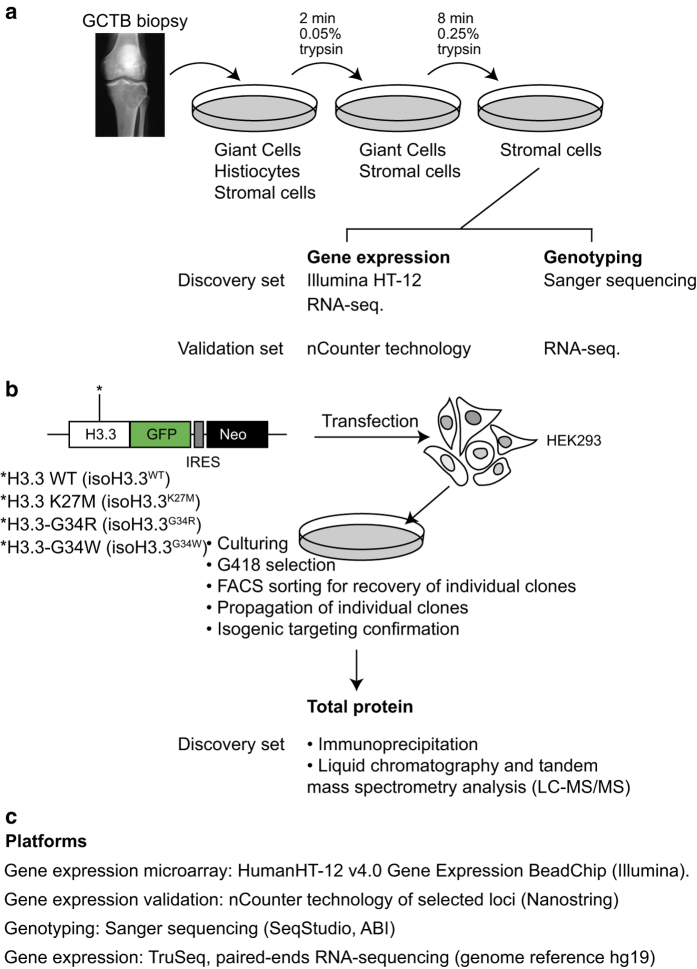
Description of the Giant Cell Tumor of the Bone sample collection procedure engineered H3.3 isogenic cell lines and performed analysis. (**a**) Depiction of the procedure to generate primary cell lines by differential trypsinizations. (**b**) From purified stromal cells, genomic DNA (gDNA) and total RNA were extracted. The discovery and validation set of samples were non-overlapping. (**c**) Isogenic cell lines generated with zinc finger targeting methodology contain H3.3 WT construct (as unmutated control), K27M, G34R, or G34W, and denoted isoH3.3^WT^, isoH3.3^K27M^, isoH3.3^G34R^, and isoH3.3^G34W^, respectively. After validation of proper H3F3A gene targeting and GFP expression analysis, GFP immunoprecipitations were performed with each of the four isogenic cell lines. The H3.3 interaction proteomes were uncovered by LC-MS/MS analysis.

As presented here, the transcriptome and interactome from GCTB biopsies and isogenic cell lines, respectively, with mutations of H3.3 have been made available (Fig. 1). We first isolated the stromal compartment from GCTB biopsies of the tumor and establishment primary cell lines for further studies (Fig. 1a). The enriched cells were used to generate gene expression microarray data and RNA sequencing. We then generated isogenic cell lines in HEK293 cells by targeting the endogenous H3F3A locus encoding H3.3 with mutations of H3.3 fused to GFP (Fig. 1b). From total protein extracts and immunoprecipitations, we purified the proteins interacting with the H3.3 mutated constructs. This constituted the comprehensive H3.3 interactome. Together we collected data from analysis platforms with the ambition to understand the function of H3.3 in cancer (Fig. 1c), which in part has been described in a previous publication^7^.

We there reported on the comparison of unmutated and H3.3 G34W tumors and found a distinct gene expression pattern likely dictated by the single mutation in H3.3. We uncovered several known genes associated with GCTB, e.g. that RANKL is affected via the downregulation of its decoy receptor OPG, and that the entire IGFBP-family of genes appear to be downregulated^7^. These genes are all targeted by the E2F transcription factor family.

**Figure 2:**
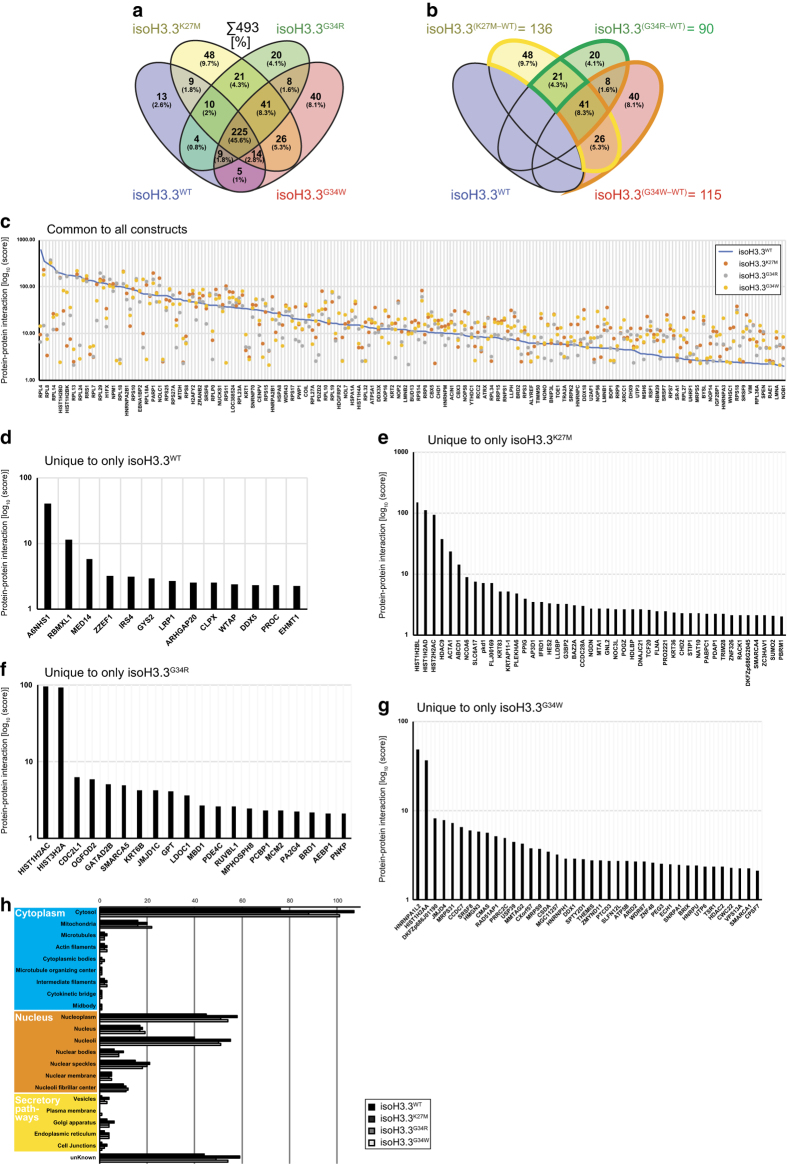
The complete H3.3 interaction proteome of the isogenic cell lines isoH3.3^WT^, isoH3.3^K27M^, isoH3.3^G34R^, and isoH3.3G34W. (**a**) Venn diagram indicating the grand total of 493 proteins in the H3.3 interactome. (**b**) Venn diagram indicating number of proteins specific to each construct when excluding isoH3.3^WT^. (**c**) Plot showing protein-protein interaction score from the H3.3 interactome when compared to isoH3.3^WT^ (blue line). (**d**-**g**) Proteins uniquely identified from the individual isoH3.3 mutant lines, with corresponding protein-protein interaction scores. (**h**) Distribution of protein compartmentalization based on proteinatlas.org subcellular location data.

We also reasoned that uncovering the interaction proteome of H3.3 in its normal and mutated form would allow us to gain further insight into its function in cancer. As previously mentioned, isogenic stable cell lines with either of the four versions of H3.3 (WT, K27M, G34R, and G34W) were established in HEK293 cells where e.g. H3.3 WT have been denoted isoH3.3^WT^ to indicate an engineered isogenic version of H3.3 to avoid confusion with patient samples. The H3.3 interaction proteome uncovered 493 proteins of which about half (225) were commonly bound by all constructs (Fig. 2a), and around 100 proteins uniquely interacted with each tested mutant of H3.3 when using WT as a reference binder (Figure 2b). While some proteins substantially loose interaction with H3.3, some also gain in binding capabilities (blue line in Figure 2c).

Our analysis in the previous study mainly focused on the H3.3 G34W substitution in GCTB, filtered against other H3.3 interaction proteomes to enrich for the once that specifically represented G34W (Figure 2a and g). This strategy successfully identified and verified the interaction of splicing-related factors (most prominently hnRNPA1L2), but all proteins identified as binding to the various mutants of H3.3 were not characterized. In this Data Descriptor we present the complete interaction proteome of H3.3 with WT versus K27M, G34R, and G34W substitutions (Table 1 and Figure 2). For a brief overview, identified proteins have been listed based on protein-protein interaction scores (Figure 2d-g). While H3.3 is a protein known to exert its function in the nucleus, the H3.3 interactomes also contain proteins from other cellular compartments (Figure 2h). We hope that the presented data will be used in conjunction with other aspects of H3.3 function in development and disease.

## Methods

Please note that samples and the generation of data presented in this Data Descriptor have been previously presented in Lim et al.^7^. While the description of the methods here is very similar to that report, we specifically want to emphasize the samples and the methods used to generate the data, in particular the previously unavailable H3.3 interaction proteome from cancer-related recurrent substitutions.

### Sample collection

The data in this data descriptor contain information generated from Giant Cell Tumor of the Bone biopsies and established control cell lines. Twenty tumor biopsies were collected from two cohorts, one from Germany and one from South Korea, to establish primary cell lines from the two clinics. Informed consent to analyse tumour tissue and to publish clinical details was obtained from all individuals included in the study. The use of patient samples and the experiments performed in this study was approved by and in accordance with guidelines and regulations by the Ethics Committees of the University of Heidelberg, University of Leipzig, University Medical Center Hamburg-Eppendorf, and the National Cancer Center of Korea (IRB NCC2015-0070).

### Transcriptome analysis (microarray and RNA-seq.)

We performed gene expression microarray analysis on 29 samples, of which 9 were German primary cell lines, 12 were Korean biopsies and 6 Korean cell lines, and 2 were control cell lines. Control cell lines were the bone-marrow derived mesenchymal stromal cell line KM1234 and the hFOB1.19 osteoblast cell line. In total there were 19 samples of H3.3 G34W and 10 were H3.3 WT. The samples were run on a HumanHT-12 v4 Expression BeadChip (Illumina). RNA sequencing was performed on 6 Korean primary cell line samples of which 3 were H3.3 G34W and 3 were H3.3 WT. We used the TrueSeq RNA kit for library preparation and paired-end sequencing on an Illumina HiSeq2500 instrument and accumulated about 14 Gbp read bases per sample with a Q30 phred quality score of 91-96%.

### Immunoprecipitation, liquid chromatography and tandem mass spectrometry analysis (LC-MS/MS)

The GFP-expressing isogenic cell lines were used for immunoprecipitations of the H3.3 construct with interacting proteins from total protein extracts. Isogenic HEK293 cells were grown in DMEM media with 10% FBS in a Ø10 cm dish, harvested at subconfluency by cell scraping, rinsed twice with PBS and lyzed with 200 μl ice-cold lysis buffer, and placed at + 4°C for 30 minutes on rotation. The lysis buffer consisted of 50 mM Tris-HCl (pH7.6), 150 mM NaCl, 1% NP-40, 0.5% sodium deoxycholate, and 0.1% SDS, supplemented with 2.5 mM MgCl_2_, 3 μl [25U/μl] Benzonase nuclease (Novagen, Millipore) and Complete protease inhibitors (Roche), Pepstatin A (Roche) and Aprotenin (Roche). Cleared lysates were diluted 1:2.5 in dilution buffer (10 mM Tris-HCl (pH7.5), 150 mM NaCl, 0.5 mM EDTA). Total protein concentrations were determined by BCA protein assays (Pierce, Thermo Scientific) and PAGE-gel followed by Coomassie (BioRad) staining to determine equal loading prior to immunoprecipitation. The equal loading-adjusted protein lysates were incubated with equilibrated 25 μl GFP-Trap-A bead slurry (Chromotek), washed and recovered with 2x Laemmli buffer (BioRad) supplemented with 10% ß-mercaptoethanol. A new PAGE-gel was run to separate the eluates, and stained with Silver stain kit (Pierce, Thermo Scientific) for verification. Each lane was divided into three equal portions and subjected to in-gel tryptic digestion and LC-MS/MS separation with the Q Exactive Hybrid Quadrupole-Orbitrap Mass spectrometer instrumentation (Thermo Fisher Scientific).[Table t2]

### Code availability

The presented data was analyzed with the following software and applications:

1. For the RNA-sequencing data, we trimmed the reads using trimmomatic0.36^8^, mapped the reads with TopHat^9^ to the human genome reference hg19, and assembled the reads with CuffLinks^10^.
2. Protein scores from the LC-MS/MS analysis were generated and analyzed with default settings using the SEQUEST package (Thermo Fisher Scientific).

## Data Records

The gene expression microarray data, generated by the Illumina HT12 array platform using total RNA from Giant Cell Tumor of the Bone biopsies, have been made available by the Gene Expression Omnibus (GEO) in Data Citation 1.

The RNA sequencing data, generated by poly-dT selected RNA from primary cell lines isolated as outlined in figure 1, have been made available by the Gene Expression Omnibus (GEO) in Data Citation 2.

The liquid chromatography and mass spectrometry (LC-MS/MS) data, generated from GFP immunoprecipitation experiments of isogenic HEK293 cell lines containing H3.3-GFP constructs, have been made available at the PRIDE Archive (www.ebi.ac.uk/pride/archive/) in line with the ProteomeXchange (PX) consortium guidelines. The public dataset (including results as mzIdentML files, peak files and raw data) is available under Data Citation 3.

Data indicating the cellular compartments of individual proteins were provided by the Human Protein Atlas (https://www.proteinatlas.org/about/download).

## Technical Validation

In a previous report utilizing the data presented here, we validated our findings with orthogonal methods^7^. First, the gene expression microarray data from GCTB biopsies (Data Citation 1) were validated with deep sequencing technology of poly-dT selected RNA from primary cell lines (Data Citation 2) originating from an independent set of samples, not overlapping with samples used to generate the microarray data (Table 1). Hierarchical clustering and GO-term analysis indicated very similar transcriptional profiles and gene functional characteristics, suggesting that the two methods were congruent^7^.

**Figure 3:**
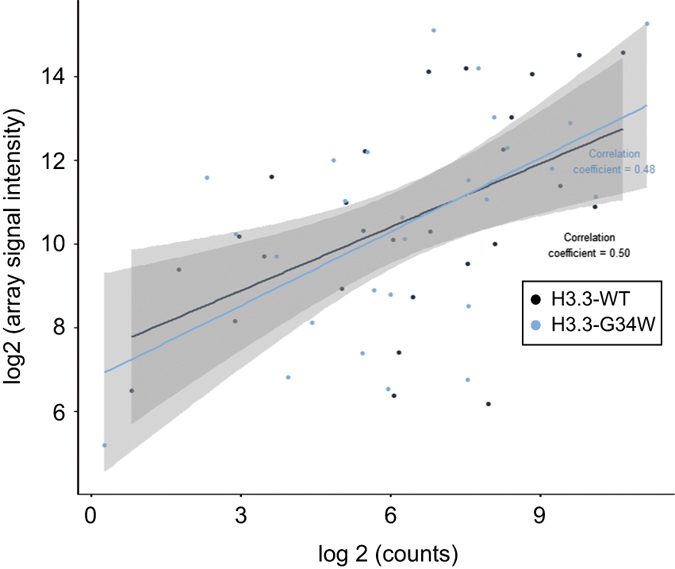
Correlation plot between two orthogonal gene expression methods using Giant Cell Tumor of the Bone biopsy RNAs. Gene expression microarray log2 signal intensity was plotted on the y-axis against data from the quantitative and hybridization-based transcript-counting nCounter technology (NanoString Tech.) on the x-axis. The Giant Cell Tumor of the Bone biopsies stem from two independent, non-overlapping samples sets. The pairwise plot indicates a strong correlation coefficient of 0.50 for H3.3^WT^ and 0.48 for H3.3^G34W^.

Second, we performed validation of the gene expression data with the RNA hybridization-based nCounter assay (NanoString Technologies). We found a strong correlation between the two methods (Figure 3; correlation coefficient 0.5 for H3.3^WT^ and 0.48 for H3.3^G34W^).

To uncover potential proteins binding the cancer-related mutated N-terminal tail of H3.3, we first generated and validated proper endogenous gene targeting of the isogenic cell lines by PCR and Southern blot analysis^7^. After confirming the GFP-expression of selected clones, we performed immunoprecipitations (IP) of the H3.3-GFP fusion proteins to uncover the H3.3 interactome, with emphasis on proteins that specifically interacted with the mutated forms. Mock IP with parental control did not pull down any proteins after the washing procedures. From the H3.3 interactome, we found hnRNPA1L2 to be the strongest interactor to H3.3^G34W^ and validated the mass spectrometry data with PAGE and Western blot analysis ^7^. Here, we make all data available to allow further analysis of expression data and the WT and mutant H3.3 interaction proteomes (Table 1).

## Additional information

**How to cite this article**: Lim, J. *et al*. Transcriptome and protein interaction profiling in cancer cells with mutations in histone H3.3. *Sci. Data*. 5:180283 doi: 10.1038/sdata.2018.283 (2018).

**Publisher’s note**: Springer Nature remains neutral with regard to jurisdictional claims in published maps and institutional affiliations.

## Supplementary Material



## Figures and Tables

**Table 1:** Summary of the protocols and proteomics data sets of isogenic cell lines containing H3.3 with WT, K27M, G34R, or G34W substitutions.

Subjects	Data partitions	Protocol 1	Protocol 2	Protocol 3	Data
isoH3.3^WT^ HEK293	Lindroth_01	Total protein extract	GFP Immuno-precipitation	Q-Exactive LC-MS/MS	PRIDE PXD009966
·/·	Lindroth_02	·/·	·/·	·/·	·/·
·/·	Lindroth_03	·/·	·/·	·/·	·/·
isoH3.3^K27M^ HEK293	Lindroth_04	Total protein extract	GFP Immuno-precipitation	Q-Exactive LC-MS/MS	PRIDE PXD009966
·/·	Lindroth_05	·/·	·/·	·/·	·/·
·/·	Lindroth_06	·/·	·/·	·/·	·/·
isoH3.3^G34R^ HEK293	Lindroth_07	Total protein extract	GFP Immuno-precipiation	Q-Exactive LC-MS/MS	PRIDE PXD009966
·/·	Lindroth_08	·/·	·/·	·/·	·/·
·/·	Lindroth_09	·/·	·/·	·/·	·/·
isoH3.3^G34W^ HEK293	Lindroth_10	Total protein extract	GFP Immuno-precipiation	Q-Exactive LC-MS/MS	PRIDE PXD009966
·/·	Lindroth_11	·/·	·/·	·/·	·/·
·/·	Lindroth_12	·/·	·/·	·/·	·/·

**Table 2 t2:** Patient Giant Cell Tumor of the Bone biopsies and biological replicates of transcriptomics data.

Source	Provenience	Samples	Genotype	Protocol 1	Protocol 3	Data
GCTB primary cell line	NCC (Rep. of Korea)	GCTB07	H3F3A (H3.3^G34W^)	RNA extraction	RNA sequencing	GSM2773985
GCTB primary cell line	NCC (Rep. of Korea)	GCTB08	H3F3A (H3.3^WT^)	RNA extraction	RNA sequencing	GSM2773986
GCTB primary cell line	NCC (Rep. of Korea)	GCTB09	H3F3A (H3.3^WT^)	RNA extraction	RNA sequencing	GSM2773987
GCTB primary cell line	NCC (Rep. of Korea)	GCTB10	H3F3A (H3.3^G34W^)	RNA extraction	RNA sequencing	GSM2773988
GCTB primary cell line	NCC (Rep. of Korea)	GCTB11	H3F3A (H3.3^WT^)	RNA extraction	RNA sequencing	GSM2773989
GCTB primary cell line	NCC (Rep. of Korea)	GCTB12	H3F3A (H3.3^G34W^)	RNA extraction	RNA sequencing	GSM2773990
MSC (control)	DKFZ (Germany)	KM1234	H3F3A (H3.3^WT^)	RNA extraction	RNA sequencing	GSM2773991
GCTB biopsy	Commercial source (ATCC)	hFOB	H3F3A (H3.3^WT^)	RNA extraction	Microarray hybridization	GSM2730210
MSC (control)	DKFZ (Germany)	KM1234	H3F3A (H3.3^WT^)	RNA extraction	Microarray hybridization	GSM2730211
GCTB biopsy	NCC (Rep. of Korea)	C05	H3F3A (H3.3^WT^)	RNA extraction	Microarray hybridization	GSM2730224
GCTB biopsy	NCC (Rep. of Korea)	C06	H3F3A (H3.3^G34W^)	RNA extraction	Microarray hybridization	GSM2730225
GCTB biopsy	NCC (Rep. of Korea)	C07	H3F3A (H3.3^G34W^)	RNA extraction	Microarray hybridization	GSM2730226
GCTB biopsy	NCC (Rep. of Korea)	C08	H3F3A (H3.3^WT^)	RNA extraction	Microarray hybridization	GSM2730227
GCTB biopsy	NCC (Rep. of Korea)	C09	H3F3A (H3.3^WT^)	RNA extraction	Microarray hybridization	GSM2730228
GCTB biopsy	NCC (Rep. of Korea)	C10	H3F3A (H3.3^G34W^)	RNA extraction	Microarray hybridization	GSM2730229
GCTB biopsy	NCC (Rep. of Korea)	WF1-2	H3F3A (H3.3^WT^)	RNA extraction	Microarray hybridization	GSM2730212
GCTB biopsy	NCC (Rep. of Korea)	WM1-6	H3F3A (H3.3^WT^)	RNA extraction	Microarray hybridization	GSM2730213
GCTB biopsy	NCC (Rep. of Korea)	WF1-9	H3F3A (H3.3^WT^)	RNA extraction	Microarray hybridization	GSM2730214
GCTB biopsy	NCC (Rep. of Korea)	GM1-15	H3F3A (H3.3^G34W^)	RNA extraction	Microarray hybridization	GSM2730215
GCTB biopsy	NCC (Rep. of Korea)	GF1-16	H3F3A (H3.3^G34W^)	RNA extraction	Microarray hybridization	GSM2730216
GCTB biopsy	NCC (Rep. of Korea)	GM1-17	H3F3A (H3.3^G34W^)	RNA extraction	Microarray hybridization	GSM2730217
GCTB biopsy	NCC (Rep. of Korea)	GM1-21	H3F3A (H3.3^G34W^)	RNA extraction	Microarray hybridization	GSM2730218
GCTB biopsy	NCC (Rep. of Korea)	GF1-22	H3F3A (H3.3^G34W^)	RNA extraction	Microarray hybridization	GSM2730219
GCTB biopsy	NCC (Rep. of Korea)	GM2-1	H3F3A (H3.3^G34W^)	RNA extraction	Microarray hybridization	GSM2730220
GCTB biopsy	NCC (Rep. of Korea)	GF2-5	H3F3A (H3.3^G34W^)	RNA extraction	Microarray hybridization	GSM2730221
GCTB biopsy	NCC (Rep. of Korea)	WM2-6	H3F3A (H3.3^WT^)	RNA extraction	Microarray hybridization	GSM2730222
GCTB biopsy	NCC (Rep. of Korea)	GF2-7	H3F3A (H3.3^G34W^)	RNA extraction	Microarray hybridization	GSM2730223
GCTB biopsy	DKFZ (Germany)	dkfzC-AO	H3F3A (H3.3^WT^)	RNA extraction	Microarray hybridization	GSM2730201
GCTB biopsy	DKFZ (Germany)	dkfzC-AP	H3F3A (H3.3^G34W^)	RNA extraction	Microarray hybridization	GSM2730202
GCTB biopsy	DKFZ (Germany)	dkfzC-AQ	H3F3A (H3.3^G34W^)	RNA extraction	Microarray hybridization	GSM2730203
GCTB biopsy	DKFZ (Germany)	dkfzC-AR	H3F3A (H3.3^G34W^)	RNA extraction	Microarray hybridization	GSM2730204
GCTB biopsy	DKFZ (Germany)	dkfzC-AS	H3F3A (H3.3^G34W^)	RNA extraction	Microarray hybridization	GSM2730205
GCTB biopsy	DKFZ (Germany)	dkfzC-AT	H3F3A (H3.3^G34W^)	RNA extraction	Microarray hybridization	GSM2730206
GCTB biopsy	DKFZ (Germany)	dkfzC-AU	H3F3A (H3.3^G34W^)	RNA extraction	Microarray hybridization	GSM2730207
GCTB biopsy	DKFZ (Germany)	dkfzC-AW	H3F3A (H3.3^G34W^)	RNA extraction	Microarray hybridization	GSM2730208
GCTB biopsy	DKFZ (Germany)	dkfzC-AX	H3F3A (H3.3^G34W^)	RNA extraction	Microarray hybridization	GSM2730209
